# A Fully Automated Radiosynthesis of [^18^F]Fluoroethyl-Diprenorphine on a Single Module by Use of SPE Cartridges for Preparation of High Quality 2-[^18^F]Fluoroethyl Tosylate

**DOI:** 10.3390/molecules18067271

**Published:** 2013-06-20

**Authors:** Bent W. Schoultz, Brian J. Reed, János Marton, Frode Willoch, Gjermund Henriksen

**Affiliations:** 1 Department of Chemistry, University of Oslo, PO Box 1033, Blindern, N-0315 Oslo, Norway; E-Mail: b.j.reed@medisin.uio.no; 2 Institute of Basic Medical Sciences, University of Oslo, PO Box 1110 Blindern, N-0317 Oslo, Norway; E-Mails: frode.willoch@medisin.uio.no (F.W.); gjermund.henriksen@petsenteret.no (G.H.); 3 ABX GmbH, Heinrich-Glaeser-Strasse 10-14, D-01454 Radeberg, Germany; E-Mail: marton@abx.de; 4 Norwegian Medical Cyclotron Centre, P.O Box 4950, Nydalen, N-0424 Oslo, Norway

**Keywords:** 2-[^18^F]fluoroethyl tosylate, ^18^F-fluoroalkylation, PET, opioid receptors, automation, solid phase extraction

## Abstract

We have developed a new method for automated production of 2-[^18^F]fluoroethyl tosylate ([^18^F]FETos) that enables ^18^F-alkylation to provide PET tracers with high chemical purity. The method is based on the removal of excess ethylene glycol bistosylate precursor by precipitation and subsequent filtration and purification of the filtrate by means of solid phase extraction cartridges (SPE). The method is integrated to a single synthesis module and thereby provides the advantage over previous methods of not requiring HPLC purification, as demonstrated by the full radiosynthesis of the potent opioid receptor PET tracer [^18^F]fluoroethyldiprenorphine.

## 1. Introduction

Meeting the demand for increased availability of PET tracers for preclinical and clinical imaging is dependent on their radiosynthesis to be performed in a fully automated approach. Hence, simplification of production methods and their adaptation to automated radiosynthesis are current priorities in radiopharmaceutical chemistry. When feasible, direct nucleophilic ^18^F-fluorination is preferred, but its applicability can be hampered by the presence of H-acidic functions in the precursors used for radiolabelling, such as e.g., Lewis bases, hydroxy-, amine-, amido- and thio-moieties. In those cases where direct labeling is not feasible, a commonly used strategy relies on the use of secondary precursors for ^18^F-fluoroalkylation. These agents are used for addition of ^18^F-fluoroalkyl moieties on precursors containing *O*-, *N-*, and *S-*atoms [1]. Importantly, the use of secondary labeling agents for ^18^F is an attractive method for making direct use of precursors that are already available for production of PET tracers by means of ^11^C-methylation [2].

Commonly used secondary labeling agents for ^18^F-fluoroalkylations include ^18^F-fluoro-(CH_3_)_n_X (n = 1–3) were X is arylsulfonates (e.g., *O*-mesyl, *O*-nosylate, *O*-tosylate or *O*-triflate) [3], or halides (bromide or iodide) [4]. These agents provide a range of reactivity both towards the initial reaction with [^18^F]fluoride and the subsequent reaction with the nucleophilic moiety contained in the precursor. 

2-[^18^F]fluoroethyl tosylate ([^18^F]FETos) [5] has gained increased relevance due to its beneficial properties for use as a precursor to perform ^18^F-fluoroalkylations: its relatively low volatility, high chemical stability and a good balance between reactivity of the tosylate leaving group, chemo-selectivity and hydrolytic stability. A previous impediment to the broad applicability of [^18^F]FETos is the frequently encountered low chemical purity resulting from classical methods for solid phase extraction (SPE), *i.e.*, a low efficacy of removal of excess ethylene glycol bistosylate precursor. This introduces a risk for cross linking of the substrate, as well as the possibility of other alkylation products. Overall, this results in low chemical purity and apparent low SA, e.g., by hydroxy-ethylated side products which can be carried along with the final ^18^F-fluoroalkylated product. This fact severely restricts the applicability of [^18^F]FETos for production of PET tracers that require a high purity, specifically for high affinity tracers directed at saturable targets, such as G-protein coupled receptors. These restrictions have so far demanded an intermediate purification of [^18^F]FETos by means of a separate HPLC method. Since several PET tracers still rely on a final purification by HPLC, these factors have prevented the implementation of a two-pot, three-step process in an automated radiosynthesis starting from [^18^F]fluoride [6,7].

Here we describe a method for purification of the intermediate [^18^F]FETos and preparation for subsequent ^18^F-fluoroalkylation by use of SPE cartridges. The method was demonstrated applicable for a fully automated radiosynthesis of [^18^F]fluoroethyldiprenorphine ([^18^F]FDPN), starting from [^18^F]fluoride.

## 2. Results and Discussion

### 2.1. Chemistry

#### 2.1.1. Radiolabelling and Purification of [^18^F]FETos

Previous methods applied for purification of [^18^F]FETos with reverse-phase SPE cartridges have been limited to the removal of [^18^F]fluoride, phase-transfer catalysts, salts and solvents, resulting in a less than optimal chemical purity of the [^18^F]FETos. One explanation for this fact may be the limiting mass capacity of small SPE cartridges for preparative work-up of reaction mixtures. The relatively high amount of precursor (10–20 mg) normally used to ensure a high radiochemical yield of [^18^F]FETos can lead to low separation efficacy and plug elusion resulting in poor product purity.

In initial experiments, we employed a test solution of FETos standard containing a lowered amount (approx. 0.5 mg) of precursor. We optimized chromatographic conditions for reversed phase C18-SPE cartridges with 35 vol. % MeOH in water, and identified a high and suitable separation factor for FETos and ethylene glycol bistosylate. Based on these results we prospected that the synthesis and purification of [^18^F]FETos could be accomplished if the excess of precursor could be removed prior to the previously established system for SPE purification. Hence our strategy to maximize yield and purity of [^18^F]FETos was to maintain the required amount of the precursor (7.5 mg in 1 mL) during the [^18^F]fluoride incorporation reaction, and reduce the precursor amount after the reaction to a mass level suitable for subsequent product isolation with the identified protocol for SPE separation. The reduction of the amount of ethylene glycol bistosylate was successfully performed by a selective precipitation of the relatively more lipophilic precursor after addition of a 3-fold volume (3 mL) of weak acetic acid (to limit hydrolysis of [^18^F]FETos) relative to that of the reaction mixture at room temperature. The bulk precipitate (>95% of the total amount) could then be removed from the polar solvent after passing the crude mixture through a 0.45 μm filter disk. The filtrate, containing >95% of the available [^18^F]FETos was directly transferred for purification by SPE. Subsequent elution of the SPE-cartridge with 35% methanol selectively mobilized [^18^F]FETos, whilst the precursor was retained on the SPE-cartridge.

#### 2.1.2. Production Runs

A representative production run of [^18^F]FETos started with 10–30 GBq [^18^F]Fluoride and gave a preparative isolated yield > 45% with a radiochemical purity > 99% (radio-TLC). Assessment of the product quality by HPLC revealed a specific activity 100–400 GBq/μmol with a reduction of ethylene glycol bistosylate by a factor > 1.5 × 10^5^ based on the limit of detection (0.25 ng/µL).

**Figure 1:**
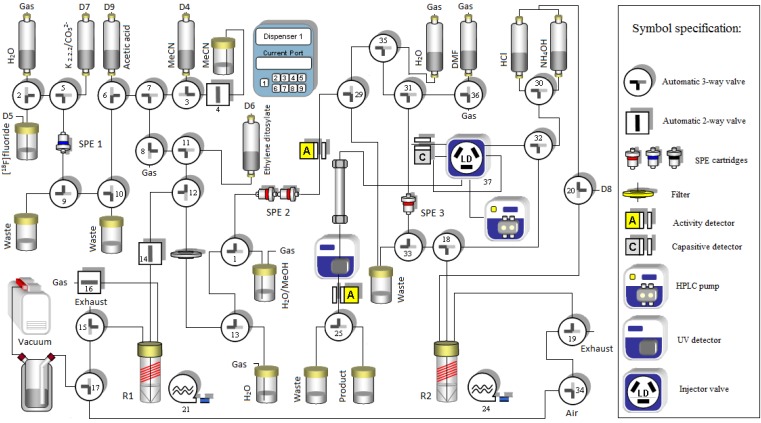
Schematic diagram showing the automated set up on a HB^III^ module (Scintomics) for the two-pot, three-step radiosynthesis; from drying of [^18^F]fluoride to HPLC separation of [^18^F]FDPN.

To encompass the ^18^F-alkylation reaction on the 3-trityl protected 6-*O*-desmethyl DPN precursor (TDDPN), highly prone to hydrolysis, the eluted product fraction was loaded onto a second SPE cartridge (Strata-X, SPE no 3, Figure 1) for product immobilization and subsequent drying by gas-purging. The resulting dry [^18^F]FETos could then quantitatively be released by elution of the cartridge to a second reactor vial for ^18^F-alkylations.

#### 2.1.3. ^18^F-Fluoroalkylation and Purification of [^18^F]Fluoroethyldiprenorphine

**Scheme 1:**
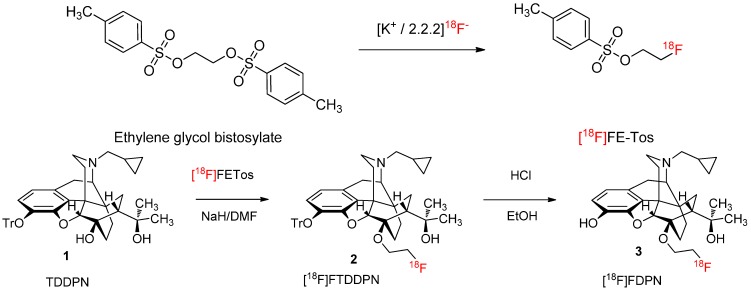
Two-pot, three-step synthesis of [^18^F]FDPN.

To evaluate the new method for isolation of [^18^F]FETos in productions involving ^18^F-fluoralkylation, we compared the quality and performance with that of the established method for production of [^18^F]fluoroethyldiprenorphine ([^18^F]FDPN) [7,8,9,10], relying on purification of [^18^F]FETos by HPLC. We used the commercially available 3-trityl protected 6-*O*-desmethyl DPN precursor (TDDPN), the established precursor for ^18^F-fluoroethylation, in a fully automated two-pot, three-step radiosynthesis. All process steps, including initial drying of [^18^F]fluoride from cyclotron target water, direct ^18^F-fluorination and isolation of [^18^F]FETos, ^18^F-fluoroalkylation, deprotection and finally preparative HPLC for product isolation of [^18^F]FDPN (Scheme 1) was successfully implemented to a HB^III^ module (Scintomics GmbH, Lindach, Germany).

All steps in the radiosynthesis of [^18^F]FDPN were automated, except for the manual addition and removal of remaining NaH that is employed for activating the 6-*O* hydroxyl moiety of the 3-trityl protected 6-*O*-desmethyl precursor. The activated precursor could then be reacted with the anhydrous solution of [^18^F]FETos in DMF in the second reactor vial. The reaction with [^18^F]FETos proceeded in high yield even with the low-reactivity nucleophile (tertiary, sterically hindered alcohol) contained in TDDPN. The radiochemical yield of the ^18^F-fluoroalkylation reaction was reproducibly >95% (n = 30). To prevent precipitation of the orvinol substrate during the acidic deprotection, we used 0.5 mL of 2 M solution of HCl in EtOH, as reported previously [11]. Subsequently, ammonium hydroxide (0.53 mL, 2 M) was added to obtain a slightly basic pH before transferring the solution to the HPLC. From the HPLC, the collected product fraction was diluted with water to facilitate trapping of [^18^F]FDPN on a reverse-phase SPE. After washing the cartridge with water (10 mL), the product was eluted with ethanol and diluted with PBS to obtain an injectable solution.

**Figure 2:**
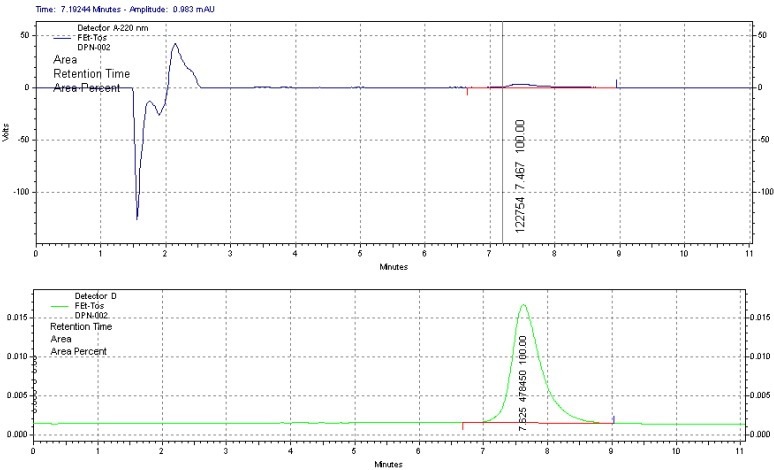
HPLC chromatogram from quality control of formulated [^18^F]FDPN. Upper part shows FDPN measured by UV and lower part shows [^18^F]FDPN measured radiometrically.

Starting from 10–30 GBq of [^18^F]fluoride, [^18^F]FDPN was produced in an decay corrected isolated radiochemical yield of 25 ± 7% (n = 30) in a synthesis time of ~100 min, in a radiochemical and chemical purity of >99% (Figure 2).

The specific activity of the product at end-of-synthesis was in the range of 50–200 GBq/μmol depending on the SA of the starting [^18^F]fluoride activity. This demonstrates that the isolation of [^18^F]FETos with precipitation of precursor combined with SPE purification yielded a similar quality of [^18^F]FDPN compared to the previous HPLC purification method [7,8,9,10].

## 3. Experimental

### 3.1. General Methods

Materials: [^18^F]fluoride was produced by the ^18^O(*p*, *n*)^18^F nuclear reaction. TDDPN, purity > 95%, was obtained from ABX GmbH (Advanced Biochemical Compounds, Radeberg, Germany).

### 3.2. Radiolabelling

The automated production protocol started by trapping and washing of [^18^F]fluoride from the cyclotron target water on an equilibrated QMA-cartridge (Sep-Pak light, Waters, Kjeller, Norway) before eluting [^18^F]fluoride to the reactor vial with a solution of acetonitrile (350 µL) with 20% water (v/v) containing Kryptofix-2.2.2. (29 µmol) and potassium carbonate (14 µmol). Azeotropic drying of the [K^+^ ⊂ 2.2.2]^18^F^−^ complex was performed under reduced pressure (200 mbar) at 75 °C with repeated exchange of the atmosphere with argon. Addition of acetonitrile (0.5 mL) and subsequent evaporation was repeated twice. The nucleophilic [^18^F]fluoride substitution was performed by the addition of ethylene glycol bistosylate (7.5 mg, purified by recrystallization in absolute EtOH) in acetonitrile (1 mL) to the reactor vial which contained the dried [K^+^ ⊂ 2.2.2 ]^18^F^−^. The reaction was allowed to proceed for 5 min at 75 °C before cooling to room temperature with compressed air. The ethylene glycol bistosylate precursor was precipitated from the reaction mixture by addition of 0.007 M acetic acid (3 mL). The resulting precipitate was allowed to settle for one minute, before the mixture was transferred to the two stacked Sep-Pak PlusC-18 cartridges (Waters, Kjeller, Norway) via a 0.45 µm polypropylene filter disk (Whatman Puradisc, 25 mm diameter, Sigma Aldrich, Norway). [^18^F]FETos immobilized on the cartridge was washed with water (2 mL) before purging with N2(g). Elution of [^18^F]FETos was performed with 35% (v/v) methanol/water, while continuously monitoring the cartridge outlet for radioactivity. The product fraction, eluting in the interval 7–17 mL, was directed on to 30 mg reversed-phase Strata-X column (Phenomenex, Værløse, Denmark). The [^18^F]FETos was retained on the cartridge, and the cartridge was subsequently flushed with argon while heating at 50 °C for 20 min. Anhydrous [^18^F]FETos was mobilised from the Strata-X column with anhydrous DMF (200 µL) and led into the second reactor for ^18^F-fluoroalkylation.

### 3.3. ^18^F-Fluoroalkylation of 3-O-Trityl-6-O-desmethyl-Diprenorphine

TDDPN (2.0 mg) was dissolved in anhydrous DMF (200 µL) and treated with dry NaH (5 mg) for 5 min. The excess solid NaH was removed, and the supernatant was added to the [^18^F]FETos. The reaction mixture was heated to 100 °C for 10 min. After this, the reactor was cooled to 40 °C, and 2.0 M HCl in EtOH (0.5 mL) was added for removal of the trityl protecting group (5 min, 40 °C). In preparation for the HPLC, the reaction mixture was brought to pH 8 by adding of 2.0 M ammonium hydroxide (0.53 mL) before transfer to the injection loop. Separation was performed by means of a Chromolith Performance RP-18, 100 length × 10 mm internal diameter (Merck, VWR, Oslo, Norway), mobile phase 35% acetonitrile and 0.1 M ammonium formate (^v^/_v_) at 5 mL/min. The fraction containing [^18^F]FDPN was isolated by radioactive peak collection by HPLC (LC, Scintomics GmbH) interfaced to the HB^III^ module. The collected product fraction, 5–10 mL, was diluted with water to obtain a concentration of acetonitrile below 20%, to facilitate immobilisation on a Sep-Pak Light cartridge. The cartridge was washed with water (10 mL) before the product was eluted by 96% EtOH (100–300 µL). The final formulation was performed by dilution with physiological phosphate buffer saline, to pH 7–8, and 0.22 µm sterile filtering (Millex, Sigma-Aldrich, St. Louis, MO, USA).

### 3.4. Quality Control

Product identification and quantitative analyses were performed on an HPLC instrument (Shimadzu, Bergman AS, Lillestrøm, Norway) equipped with a UV detector, set at 220 nm, and a GABI radioactivity detector (Raytest GmbH, Straubenhardt, Germany). The analytical system was a µ-Bondapak C-18 HPLC column 300 length × 4 mm internal diameter (CS-Chromatographie GmbH, Langerwehe, Germany) eluted with 50:50 (^v^/_v_) acetonitrile:0.1 M ammonium formate at a flow rate of 1.5 mL/min. The identity of radiolabelled products was performed by comparison of retention values for authentic samples of FDPN and FETos (ABX Biochemicals). The SA of the formulated products was calculated by measuring the radioactivity of samples in combination with determination of the amount of cold carrier, as determined by a standard curve for UV absorbency obtained by analytical HPLC. Determination of radiochemical purity was measured by HPLC and on a radio-TLC system based on silica aluminium sheets (Silica gel 60 F254, Merck, VWR, Oslo, Norway) with a mobile phase consisting of 4:1 (^v^/_v_), acetonitrile:0.1 M ammonium formate. Eluted radioactive fractions were spotted by a mini Gita radio-TLC scanner (Raytest) with R_f_-values: [^18^F]fluoride: 0, [^18^F]FETos: 0.88, [^18^F]FDPN: 0.56. Retention times with HPLC for [^18^F]fluoride was 2–3 min, ethylene glycol bistosylate: 13.1 min, [^18^F]FETos: 6.9 min, [^18^F]FTDDPN: 9.3 min and [^18^F]FDPN: 7.5 min.

## 4. Conclusions

Our methodology based on reducing the amount of ethylene glycol bistosylate precursor by precipitation enables SPE purification of [^18^F]FETos on a production-relevant radioactivity level. The practical utility and the performance of the new method for isolation and purification of [^18^F]FETos was demonstrated in a fully automated two-pot, three-step radiosynthesis of [^18^F]FDPN, resulting in a quality and yield comparable to previously reported methods. This new and simplified method improves the availability of PET tracers based on [^18^F]FETos for automated routine production.

## Abbreviations

PET: positron emission tomography
SPE: solid phase extraction
HPLC: high performance liquid chromatography
[^18^F]FETos: 2-[^18^F]fluoroethyl tosylate
[^18^F]FDPN: [^18^F]fluoro-ethyldiprenorphine
TDDPN: 3-*O*-trityl-6-*O*-desmethyldiprenorphine
HB^III^: Hot Box III, synthesis module
TLC: thin layer chromatography
PBS: phosphate buffered saline
